# The complete mitochondrial genome of *Sapajus flavius*（Blonde Capuchin）

**DOI:** 10.1080/23802359.2019.1662748

**Published:** 2019-09-12

**Authors:** Zhaonan Hao, Cao Yi

**Affiliations:** Microbiology and Metabolic Engineering of Key Laboratory of Sichuan Province, College of Life Sciences, Sichuan University, Chengdu, China

**Keywords:** *Sapajus flavius*, mitochondrial genome, protein-coding genes, phylogenetic

## Abstract

*Sapajus flavius* has been listed on the International Union for Conservation of Nature (IUCN) Red List of Threatened Species. The complete mitochondrial genome sequence of *Sapajus flavius* is presented here first, sequenced by next-generation sequencing (NGS). The *Sapajus flavius* mitogenome is 16,543 bp long, contains 13 protein-coding genes (PCGs), 2 rRNA genes (12S rRNA and 16S rRNA), 22 transfer RNA (tRNA) genes, and one control region (D-loop). The complete mitochondrial genome sequence provided here could help in the study of ecological and evolutionary research of *Sapajus* and conservation genetics of *S .flavius*.

The Blonde Capuchin, belongs to *Sapajus*, lived in lowland coastal rain forest and Montrichiardia linina swamp in north-east Brazil, secondary forest, semi-deciduous seasonal forest (Pontes et al. 2006), is on the IUCN Red List of Threatened Species (de Oliveira et al. 2015). Due to housing, hunting, trapping, and other human activities, the populations of this species are decreasing. Up to now, classifying this species was difficult by morphology and the molecular studies are limited. To know more about the biological diversity of this species and protect them, we assembled the mitochondrial genome of *Sapajus flavius.*

The raw reads of whole genome sequencing from the Blonde Capuchin’s muscle (Accession no. SAMN08637943, specimen voucher: CPB:475, 7°01′S, 34°96′W Brazil), sequenced by Illumina HiSeq 2500, has been used (SRR6811862) and then were trimmed by Trimmomatic v0.4.0 (Bolger et al. 2014), assembled and annotated with NOVOPlasty v2.7.2 (Dierckxsens et al. 2016) and MITOS2 (Bernt et al. 2013), respectively. We used *Sapajus xanthosternos* (NC_021961.1) as the reference during assembly. Finally, we got 16,543 bp long, double-stranded circular DNA (GenBank Accession No. MN218642). This mitogenome of *S .flavius* includes 13 protein-coding genes, 2 ribosomal RNA genes (12S rRNA and 16S rRNA), 22 tRNA genes, and 1 control region; The contents of A, T, G, and C are 32.98, 27.88, 26.47, and 12.66%. GC contents are 39.13%. All of the PCGs use complete (ATG, ATG, GTG) start codon and among them, 10 of the PCGs have complete stop codon (TAA, TAG, AGG), which also proves the integrity of our assembly. The lengths of 12S rRNA and 16S rRNA genes are 960 and 1,554 bp, respectively. The length of 22 tRNA genes ranges from 60 bp (tRNA-Ser) to 75 bp (tRNA-Leu). The D-loop is 185 bp and lies between the tRNA-Phe and tRNA-Pro.

Phylogenetic analysis of 14 mitogenomes using RAxML v8.2.7 with maximum likelihood (ML) method (Stamatakis 2014). *Bos taurus* was used as an outgroup. As it turns out that *Sapajus flavius* is closest to *Sapajus xanthosternos,* basically consistent with the existing research (Finstermeier et al. 2013; Lima et al. 2018) (Figure. 1). The mitogenome *Sapajus flavius* provides useful resources to study the phylogeny and evolution of Sapajus.

**Figure 1.**
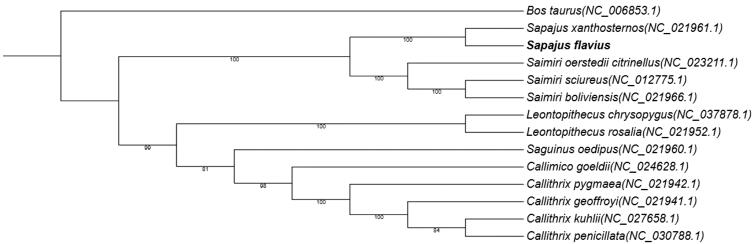
Phylogenetic tree constructed with *Sapajus flavius* and 13 other species mitogenomes. It was constructed based on the alignment of MUSCLE v3.8.425 (Edgar 2004). The bootstrap support values are generated using 100 replications.
